# The Role of Invariant NKT in Autoimmune Liver Disease: Can Vitamin D Act as an Immunomodulator?

**DOI:** 10.1155/2018/8197937

**Published:** 2018-06-26

**Authors:** Daniel S. Smyk, Athanasios Mavropoulos, Giorgina Mieli-Vergani, Diego Vergani, Marco Lenzi, Dimitrios P. Bogdanos

**Affiliations:** ^1^Institute of Liver Studies, King's College London, Faculty of Life Sciences & Medicine at King's College Hospital, Denmark Hill, London, UK; ^2^Department of Laboratory Medicine and Pathology, Faculty of Medicine, University of Alberta, Edmonton, AB, Canada; ^3^Department of Rheumatology and Clinical Immunology, Faculty of Medicine, School of Health Sciences, University of Thessaly, Biopolis, Larissa, Greece; ^4^Paediatric Liver, GI &Nutrition Centre, MowatLabs, King's College London, Faculty of Life Sciences & Medicine at King's College Hospital, Denmark Hill, London, UK; ^5^Department of Clinical Medicine, University of Bologna, Bologna, Italy

## Abstract

Natural killer T (NKT) cells are a distinct lineage of T cells which express both the T cell receptor (TCR) and natural killer (NK) cell markers. Invariant NKT (iNKT) cells bear an invariant TCR and recognize a small variety of glycolipid antigens presented by CD1d (nonclassical MHC-I). CD1d-restricted iNKT cells are regulators of immune responses and produce cytokines that may be proinflammatory (such as interferon-gamma (IFN-*γ*)) or anti-inflammatory (such as IL-4). iNKT cells also appear to play a role in B cell regulation and antibody production. Alpha-galactosylceramide (*α*-GalCer), a derivative of the marine sponge, is a potent stimulator of iNKT cells and has been proposed as a therapeutic iNKT cell activator. Invariant NKT cells have been implicated in the development and perpetuation of several autoimmune diseases such as multiple sclerosis and systemic lupus erythematosus (SLE). Animal models of SLE have shown abnormalities in iNKT cells numbers and function, and an inverse correlation between the frequency of NKT cells and IgG levels has also been observed. The role of iNKT cells in autoimmune liver disease (AiLD) has not been extensively studied. This review discusses the current data with regard to iNKT cells function in AiLD, in addition to providing an overview of iNKT cells function in other autoimmune conditions and animal models. We also discuss data regarding the immunomodulatory effects of vitamin D on iNKT cells, which may serve as a potential therapeutic target, given that deficiencies in vitamin D have been reported in various autoimmune disorders.

## 1. Introduction

Natural killer T (NKT) cells are a component of the innate immune system, which initiate and refine innate and adaptive immune responses. NKT cells can be subdivided into type 1 and type 2 NKT cells based on their T cell receptors (TCR), with type 1 NKT cells being commonly known as invariant NKT (iNKT) cells [1–3]. These two types of NKT cells display distinct roles involved in either the promotion or control of immune responses [4]. The role of iNKT cells and their mediators has been well defined in several conditions, including some autoimmune diseases such as multiple sclerosis (MS) [5]. However, the role of iNKT cells in other autoimmune conditions remains largely unexplored.

The liver's immune system is particularly specialized in dealing with exposure to dietary and commensal microbial antigens, to which it must remain tolerant. Hepatic immune tolerance is modulated by antigen-presenting cells, such as dendritic cells, Kupffer cells, hepatic stellate cells, and liver endothelial cells [6]. The role of these populations is to constantly present harmless antigens to T cells and facilitate their commitment to apoptosis, anergy, or differentiation into regulatory T cells. Still, the liver is also able to respond to pathogenic stimuli and is equipped with cellular machinery to override immune tolerance. A variety of innate lymphocytic populations, including NKT cells, *γδ* T cells, mucosal-associated invariant T cells, and CD56(+) natural killer (NK) cells are resident or can rapidly accumulate in the hepatic microenvironment following potential pathogenic challenge [7, 8]. These cells can maintain or override hepatic immune tolerance to autoantigens, leading to expansion of autoreactive T cells that mediate liver injury causing autoimmune liver disease or direct liver injury by killing hepatocytes or bile duct cells [9, 10].

Autoimmune liver diseases (AiLD) include primary biliary cholangitis (PBC), formerly known as primary biliary cirrhosis, autoimmune hepatitis (AIH), and primary sclerosing cholangitis (PSC) [28, 29]. Hepatocytes are the target of autoimmune attack in AIH, whereas the biliary epithelial cells are the targets in PBC and PSC [30]. In PBC, the small- and medium-sized bile ducts are affected, as opposed to the larger bile ducts in PSC [24]. The demographic, epidemiological, and clinical characteristics of these three conditions are distinct, and a variety of genetic [31], immunological, and environmental factors have been implicated in the disease development [32–43].

This review will examine the current knowledge regarding the role of iNKT cells in AiLD. We will first provide a general overview and update of iNKT cells function in other conditions as well as in experimental models. We will also discuss the emerging role of vitamin D in iNKT cells immunomodulation, which may serve as a therapeutic target [44–46].

## 2. Subtypes of NKT Cells and an Overview of Their Behavior

NKT cells are subdivided into type 1 (iNKT) and type 2 (NKT). iNKT cells are innate immune T cells that express the T cell receptor (TCR) V*α*24-J*α*18/V*β*11, natural killer (NK) cell surface markers (such as NK1.1, Ly149, CD161 and CD56), and activation markers CD25, CD69, and CD122 [47–51]. Liver iNKT cells have been recently shown to constitutively express the costimulatory tumor necrosis factor superfamily receptor OX40 [52]. Memory NK-like T cell populations also exist in peripheral blood such as CD8(+) T cells responding to innate IL-12 and IL-18 stimulation and coexpressing the transcription factor Eomesodermin (Eomes) and KIR/NKG2A membrane receptors [53]. Additionally, murine iNKT cells can express certain toll-like receptors (TLR), which facilitate TLR costimulation of iNKT cells in the presence of suboptimal concentrations of TCR agonists enhancing their cellular activation [54].

NKT cells recognize host and microbial lipid and/or alpha-mannosyl glycolipid antigens via CD1d (MHC class I-like molecule) through their TCR [55–57]. Activated NKT cells secrete Th1 cytokines (including IFN-*γ* and TNF-*α*), Th2 cytokines (such as IL-4 and IL-10), and Th17 cytokines (namely, IL-17 and IL-22) [58]. Hence, NKT cells play an important role in immune system regulation by polarizing Th1, Th2, Th17, and Treg cells. iNKT cells also appear to have effects on B cells, NK cells, and dendritic cells (DCs) [59–61]. Innate immunity receptors within APCs, such as DCs, activate iNKT cells through the combined presentation of lipids by CD1d and production of proinflammatory cytokines, such as IL-12 and type I IFNs [62]. Innate receptors include TLRs, Nod-like receptors (NLRs), Rig-I-like receptors (RLRs), and C-type lectin-like receptors [63].

NKT cells display both proinflammatory and anti-inflammatory behaviors, with iNKT cells generally being proinflammatory and type 2 NKT cells being suppressors of inflammation. However, recent data also indicates a possible role of type 2 NKT cells in promoting chronic inflammation [64, 65]. Hence, these roles may be reversed in differing pathological states [64]. For example, iNKT cells appear to have suppressive behavior in both experimental autoimmune encephalomyelitis (EAE) animal model and patients with MS [66, 67] but an inflammatory role in allergen-induced airway disease [60, 68–70]. iNKT cells cytokine/chemokine production has been shown to be chemoattractant to neutrophils and macrophages in organs such as the liver [9, 71]. Opposing roles for iNKT and type 2 NKT cells have been proposed in autoimmunity [72, 73]. Generally speaking, iNKT cells are believed to have a predominantly proinflammatory role [73–78], although they have also been observed to secrete IL-4 after stimulation with alpha-galactosylceramide (*α*-GalCer) [9, 71, 79]. Type 2 NKT cells (which are sulfatide-reactive [50, 51, 80]) have been shown to be anti-inflammatory and appear to inhibit iNKT cells function [9, 75]. Transfer of DCs from sulfatide-treated animals to naïve recipients resulted in iNKT cells anergy [73].

Despite some experimental studies demonstrating opposing roles for iNKT and type 2 NKT cells, several other studies have shown dual proinflammatory and anti-inflammatory roles for iNKT cells. An anti-inflammatory role has been proposed for iNKT cells in Chagas disease, whereas type 2 NKT cells were found to be inflammatory/pathogenic [81]. In a murine model of Schistosomiasis, a proinflammatory role via Th1 cytokines has been described for iNKT cells, whereas type 2 NKT cells act via a Th2 response [82]. Miyazaki et al. [83] noticed decreased levels of mucosal associated iNKT cells in the peripheral blood of patients with MS, which were especially reduced during relapses. Invariant NKT cells levels reflected disease activity with decreasing iNKT cells in MS flares [83]. Levels of iNKT cells increased with clinical recovery [83]. Other studies have reported similar findings in experimental autoimmune encephalomyelitis (EAE), whereby activating iNKT cells with *α*-GalCer modulated the disease course [84–87]. The anti-inflammatory role by iNKT cells is believed to be due to IL-4 and IL-10 secretion, which promotes a deviation to a Th2 cytokine response [87], although a role for IFN-*γ* has also been postulated [88, 89]. Other studies note protection from autoimmune disease following iNKT cells stimulation by *α*-GalCer in NOD mice [90, 91]. Invariant NKT cells have also been implicated in the progression of several autoimmune conditions, indicating differing roles of iNKT cells in various disorders [8, 24, 92–94].

## 3. iNKT Cells and B Cell Regulation

Current research indicates a regulatory role of iNKT cells over B cells, which is dependent on the interaction of iNKT cells with B cells via CD1d, which may be IL-4-driven [95–98]. It appears that iNKT cells are recruited to activate B cells in response to antigenic lipids, thus enhancing antibody response [99]. Recent studies in mice, however, have shown that iNKT cells stimulation culminated in the rapid activation and hepatic recruitment of innate-like regulatory B cells [100, 101]. Regulatory B cells (Bregs) influence immune responses primarily, although not exclusively, via the production of IL-10. The importance of human Bregs in the maintenance of immune homeostasis has been documented in several autoimmune-related pathologies [102–105].

It has also been found that marginal zone B cells are capable of activating iNKT cells [106, 107]. Bialecki and colleagues [106] found that marginal zone B cells sensitized with *α*-GalCer activated iNKT cells hybridomas but were unable to directly activate ex vivo sorted iNKT cells in the absence of DCs in culture. DC activation of iNKT cells was enhanced by marginal zone B cells and reduced in their absence [106]. It was also found that in vivo transfer of *α*-GalCer loaded marginal zone B cells activated both iNKT and NK cells [106].

The role of iNKT cells with regard to antibody production appears to be diverse, with some studies indicating an enhancement of antibody/autoantibody production via iNKT cells, whereas others note a reduction. This may be due to differing action of iNKT cells subtypes. It has been noted that CD1d deficiency in animal models exacerbates autoantibody production [108]. Wermeling et al. [98] injected murine models with apoptotic cells to trigger autoantibody production and found that reduced or absent iNKT cells resulted in increased autoreactive B cell activation, which was also observed in models where CD1d expression was absent on B cells. In response to injected apoptotic cells, iNKT cells upregulated the activation marker CD69, in association with decreased IFN-*γ* but increased IL-10 production [98]. However, IFN-*γ* was increased in NK cells and CD4+ T cells [98]. In splenic CD1d -/- CD45.1-B cells (GL7hi and CD95hi), IgM and IgG3 anti-DNA production was increased in association with increased survival of those B cells [98]. A second animal model with a 50% reduction in iNKT cells (J*α*18+/-) showed increased IgG anti-DNA and splenic germinal center B cell levels, and repopulation with iNKT cells resulted in decreased IgG3 anti-DNA production and a decreased percentage of germinal center B cells [98]. Yang et al. [108] reported similar findings, where iNKT cells suppressed IgG anti-DNA Ab and rheumatoid factor production but increased total IgG production and enhanced activation markers on B cells. That study also found that both autoreactive and nonautoreactive B cells were activated by iNKT cells, with autoreactive B cells expressing higher levels of CD1d [108].

Differing biological actions of iNKT cells subsets influence differential B cell function. An earlier study by Galli et al. [109] established that immunizing mice with *α*-GalCer and proteins resulted in increased antibody titers compared to immunization with protein alone and that decay of circulating antibodies occurred more rapidly in iNKT cells-deficient mice. These observations have also been noted in additional studies [110–113]. Galli et al. [96, 97] note two major iNKT subtypes: CD4+ and CD4-CD8- or double negative (DN), with CD4+ inducing higher levels of immunoglobulin production. A study by Zeng and colleagues [114] reports a CD4+CD8*α*+ subtype: coculturing CD4+CD8*α*+ iNKT and DN iNKT cells with peripheral B cells, they found that IgG, IgM, and IgA were released by B cells in the absence of *α*-GalCer [114]. CD4+ and DN iNKT cells secreted Th1 and Th2 cytokines when cultured with B cells pulsed with *α*-GalCer but at a lower level compared to iNKT cells cultured with dendritic cells [114]. CD4+ cells were also found to induce regulatory B cell expansion, in addition to increasing B cell production of IL-4 and IL-10 [114]. DN iNKT cells were found to express CD107*α* (a cytotoxic degranulation marker) when exposed to B cells [114]. In the presence of iNKT cells, B cells were unable to stimulate alloreactive conventional T cells [114]. A recent study by Tang et al. [115] examined the behavior of iNKT cells subsets based on Ly108 expression, which distinguishes iNKT cells that help B cells and secrete IL-21 from iNKT cells that secrete IL-17. Ly108^Lo^CD4-NK1.1- secreted IL-17, while Ly108^hi^CD4+NK1.1- promoted B cell secretion of IgG isotype anti-nuclear antibodies and IL-21 [115].

The above studies indicate a modulatory role for iNKT cells on B cells, which appears to both stimulate and control (auto)antibody production. This may be due to differing actions by iNKT cells subsets. The identification of these subsets and their functional phenotypes warrants further study.

## 4. iNKT Cells and MDSC/Treg Regulation

iNKT cells upon antigenic stimulation and the production of Th1 (IFN-*γ* and TNF-*α*) and Th2 (IL-4, IL-5, and IL-13) cytokines can also act through additional suppressive cell subsets such as myeloid derived suppressor cells (MDSCs) and regulatory T cells (Tregs) [116–119]. In vivo cytokine neutralization experiments have revealed a prominent role for IL-4, IL-10, and IFN-*γ* in the iNKT cells-mediated regulation of T cell lineage development such as Th17 [89]. MDSCs are abundant in liver/spleen and express higher levels of chemokine receptors such as CCR2, CX3CR1, and CXCR2 [120]. They also express CD11b and Gr-1 markers [121] and therefore encompass diverse cell subsets such as immature DCs, immature macrophages, and granulocytes [122]. In tumor-bearing mice, two main MDSC subtypes have been reported: granulocytic (G-MDSC) and monocytic (M-MDSC) [123]. In humans, MDSCs are predominantly characterized by expression of CD14, whereas G-MDSC are mainly CD15+, both being CD33+ HLA-DR− [124].

MDSCs are proficient in suppressing T cell proliferation and promoting tumor growth [125]. Both MDSC and Treg cells are major components of the hepatic immune suppressive tumor microenvironment (TME) [126, 127]. In tumor-bearing mice, large amounts of myeloid-derived suppressor cells (MDSCs) are recruited into the liver following Con-A-induced hepatitis [128]. MDSCs are essential for immune mediated suppression within the liver, as they electively reduce IFN-*γ* production from NKT cells through membrane-bound transforming growth factor-*β* (TGF-*β*) [128]. The absence of iNKT cells also markedly decreases the total number of intestinal polyps and is associated with a reduced frequency of Tregs cells and lower expression levels of FoxP3 protein and transcript uniquely in the polyps of Apc^Min/+^ mice, a model for colorectal cancer [129].

The exact mechanisms that influence the activity and interaction of iNKT cells/MDSCs in vivo remain ill-defined. However, it has been documented that CD1d-restricted NKT cells can enhance MDSC suppressive activity by secreting IL-13 [130]. IL-13 has been reported to mediate its effect via the IL-4R–STAT6 pathway and can induce TGF-*β*-producing CD11b+ Gr-1+ MDSC [130]. CD11b+ invariant NKT cells have the ability to inhibit T cell proliferation via membrane-bound TGF-*β*1 [131]. The induction of MDSC via IL-33 has been proposed as an alternative mechanism for *α*-GalCer-elicited iNKT cells-mediated immunosuppression [132].

In contrast, De Santo et al. have demonstrated that the absence of invariant NKT (iNKT) cells in mice during Influenza IAV infection resulted in the expansion of MDSCs, which suppressed IAV-specific immune responses through the expression of both arginase and NOS, resulting in high IAV titer and increased mortality [133]. Adoptive transfer of iNKT cells abolished the suppressive activity of MDSCs, restored IAV-specific immune responses, reduced IAV titer, and increased survival rate. The cross-talk between iNKT cells and MDSCs was CD1d- and CD40-dependent [133]. Ko et al. also showed that iNKT cells activated by *α*-GalCer-loaded CD11b+ Gr-1+ MDSC could convert MDSC into stimulatory APC [134]. Such reprogrammed MDSC upregulated the expression of CD11b, CD11c, and CD86. Hence, iNKT cells can acquire the ability to enhance suppression or convert immunosuppressive MDSCs into immunity-promoting antigen-presenting cells.

MDSCs are also an abundant cell subset during bone marrow cell (BMC) transfer. MDSCs are essential for iNKT cells-mediated Foxp3+ Treg cell expansion in recipient mice of transplantation tolerance [135]. NKT cells act through bone marrow-derived cells to suppress NK cell activity in the liver and exacerbate hepatic melanoma metastases [136]. The development of melanoma liver metastases was associated with upregulation of IL-10 in the liver and an elevated expression of IL-10 receptor on liver NK cells. Hepatoprotective effect of certain diet molecules such as the enzymatic isolate of soybeans DT56a was also associated with changes in NKT cells and Tregs [137]. iNKT cells are also capable of diminishing adverse autoimmune responses by increasing both total Tregs and follicular Tregs (Tfr) as shown in the cGVHD murine model that recapitulates several aspects of autoimmunity and internal organ fibrosis [138].

## 5. iNKT Cells and Autoimmune Liver Disease

Autoimmune liver diseases (AiLD) include autoimmune hepatitis (AIH), primary biliary cholangitis (PBC), formerly known as primary biliary cirrhosis, and primary sclerosing cholangitis (PSC). The aetiopathogeneses of these conditions have not been fully defined but appear to involve genetic, immunological, and environmental factors working in unison [32–43]. It is widely believed that an imbalance of proinflammatory and anti-inflammatory immune responses within the liver plays a large role in the development of AiLD, with an upregulation of proinflammatory immune responses and decreased or defective anti-inflammatory responses.

(Tregs) dysfunction also appears to play a role [139–142]. In AiLD, self-antigens are presented by antigen-presenting cells that directly or indirectly activate innate immune cells resident within the liver, which also include NKT cells [58, 143, 144]. Tissue-resident immune cells in general have a crucial role in local and systemic immune responses. The liver, in particular, can host a significant number of iNKT cells, but the mechanisms that regulate their survival and homeostasis have not been completely elucidated. Hepatocyte-specific expression of IL-15R*α* and localized availability of IL-15 are required to maintain the homeostasis of NK and NKT cells in the liver [145, 146]. Within the liver, NKT cells are mostly found in the sinusoids and are able to produce various cytokines (both proinflammatory and anti-inflammatory) [143]. NKT cells are capable of activating other innate and adaptive immune cells resident within the liver and regulate or enhance immune responses [65, 147, 148]. iNKT cells have been shown to activate hepatic stellate cells [77], and direct hepatocyte killing has been observed by iNKT cells or by NK cells stimulated by iNKT cells [9, 71]. Durante-Mangoni et al. [149] found low CD1d and iNKT cells but high CD161+CD56+ NKT cells in the healthy human liver, with an upregulation of CD1d on biliary epithelial cells next to portal tract fibrotic areas in patients with chronic HCV. Hepatic type 2 NKT cells produced large amounts of IFN-gamma and less IL-13 and IL-4 [149]. It was suggested that hepatic cells infected with HCV could increase CD1d and process CD1d liver antigens for presentation [149]. Another study reports that iNKT cells tend to localize in peripheral tissues (such as the liver) as opposed to lymphoid tissue and found that iNKT cells stimulate intrahepatic CD8 T cell effector responses to liver antigens [150].

Liver iNKT cells have also been shown to constitutively express the costimulatory TNF superfamily receptor OX40 [52]. OX40 stimulation results in massive pyroptotic death of iNKT cells, characterized by the secretion of proinflammatory cytokines that induce liver injury. The OX40/NKT pyroptosis pathway plays a fundamental role in concanavalin A-induced murine hepatitis as well. The poly(ADP-ribose) polymerase (PARP) proteins also induce cell death and inflammation. Chemical inhibition of PARP activity has been shown to be protective against liver injury during Con-A-induced hepatitis, where inflammation and induced hepatocyte death are mainly mediated by the activated iNKT cells lymphocyte population [19].

**Figure 1:**
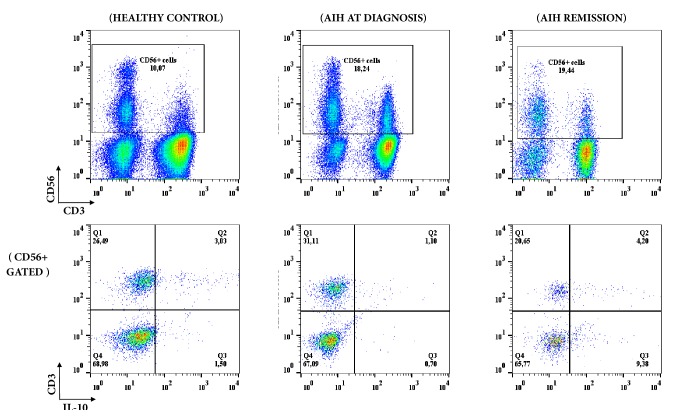
Schematic representation of phenotypic and intracellular IL-10 expression analysis in NKT (CD3+CD56+) and NK (CD3- CD56+) cells from a healthy donor and an AIH patient at diagnosis 6 months after immunosuppressive treatment, at the time of remission.

The precise role of iNKT cells in the liver during AiLD, specifically whether they are proinflammatory or anti-inflammatory, has not been fully clarified (Figure 1). Most studies are based on animal models and appear to indicate varying roles for iNKT cells in the AiLD (Table 1).

## 6. Autoimmune Hepatitis

Most studies regarding iNKT cells in AIH have been based on the murine model of Con-A-induced AIH [11, 12] and/or further genetic modifications of important signaling molecules such as PKC*θ* [18] and SOCS1, 3 [16, 17], as well as the carbon tetrachloride (CCl(4)) model of induced acute hepatitis [20, 151]. Kaneko et al. [13] found that Con-A induces hepatic NKT cells to produce IL-4, which in turn induced an increase in the expression of granzyme B and Fas ligand (FasL), promoting hepatocyte cytotoxicity. Ajuebor et al. [14] used the Con-A model to study iNKT cells function and found that Con-A activates iNKT cells, resulting in increased IL-4 and decreased IFN-*γ* production when CCL2/MCP-1 is neutralized. An interesting study by Takeda and colleagues [15] found that CD1d-deficient mice lack NKT cells and are resistant to Con-A-induced hepatitis. Transfer of NKT cells from wild-type to CD1d-deficient mice rendered them susceptible to Con-A hepatitis, an event not observed if mice were FasL-deficient [15]. Con-A administration resulted in increased FasL expression on the NKT cells surface and increased FasL-mediated cytotoxicity [15]. Similar results were reported by Biburger et al. [152] who found that *α*-GalCer enhanced TNF-*α* secretion, which in turn increased FasL expression on NKT cells. That group proposed that FasL on NKT cells interacts with Fas-expressing hepatocytes, inducing hepatocyte cell death, which raises the possibility that natural autoantigens take the place of *α*-GalCer, being presented to NKT cells by CD1d [152]. In another recent study, TPL2, a MAPKKK kinase that has also been acknowledged for its activating role in macrophage cytokine production [153], was shown to be a crucial signaling factor in iNKT cells and mediator of hepatic inflammation [21]. Genetic ablation of TPL2 ameliorated liver injury induced by Con-A without affecting NKT cells development in the thymus. The receptor-interacting protein kinase 3 (RIPK3) also plays an important role in programmed necrosis and innate inflammatory responses. Very little is known about the involvement of RIPK3 in NKT cell-mediated immune responses, but recent research has indicated that RIPK3 influences NKT cells function via activation of the mitochondrial phosphatase phosphoglycerate mutase 5 (PGAM5) [23]. PGAM5-mediated programmed necrosis of hepatocytes has been recently documented to be able to drive acute liver injury [22]. PGAM5 was highly expressed in hepatocytes of patients with AIH and in mice with Con-A-induced experimental hepatitis. Deficiency of PGAM5 protected mice from Con-A-induced hepatocellular death and liver injury. Lately, evidence has been provided to support the role of NKT cells as detectors to sense traumatic injury and to modulate the local immune response toward a restitution phase by affecting the local cytokine milieu [154]. Recently, betulin, an immunomodulatory compound extracted from* Hedyotis hedyotidea, *was shown to be able to ameliorate concanavalin-A-induced autoimmune hepatitis in mice through inhibition of NKT cells- and T cell-derived IFN-*γ*, TNF-*α*, and IL-6 cytokine expression [155]. Of interest, transient expression of transgenic IL-12 in murine liver triggered an inflammatory response mimicking human autoimmune hepatitis, where IFN-*γ* was identified as an essential mediator of liver damage, and CD4 and CD8 T cells but not NK, NKT, or B cells were essential executors of hepatic injury [156].

## 7. Primary Biliary Cholangitis

The role of iNKT cells in PBC has not been fully examined. An early study has reported increased frequency of iNKT cells in the livers of PBC patients, with a decreased number in the peripheral blood [8]. Three distinct subpopulations of iNKT cells have been noted in PBC patients so far: CD4-CD8+, CD4-CD8-, and CD4+CD8- [8]. An immunohistochemical study by Harada et al. [157] demonstrated CD3+CD57+ cells within the portal tracts and parenchyma of PBC patients and controls, with more pronounced presence of these cells within the portal tracts of PBC patients. CD3+CD57+ cells congregated around areas of injured interlobular bile ducts in PBC cases but not in healthy and pathological controls [157]. Improved detection methods have allowed accurate cytokine measurements from liver CD1d-restricted intrahepatic lymphocytes (IHL), revealing the ability to produce IFN*γ*, as well as variable levels of IL-10, IL-4, and IL-13 ex vivo [158, 159]. In murine models of PBC, iNKT cells appear to exacerbate murine autoimmune cholangitis, fibrosis, and liver injury [24–26, 160]. Infection of mice with* N. aromaticivorans* induced signature antibodies against microbial PDC-E2 and its mitochondrial counterpart but also triggered chronic T cell-mediated autoimmunity against small bile ducts [161]. Disease induction required NKT cells, which specifically respond to* N. aromaticivorans* cell wall alpha-glucuronosyl ceramides presented by CD1d molecules [161]. Mice immunized with *α*-GalCer demonstrated profound disease exacerbation with increased CD8+ T cell infiltrates, portal inflammation, granuloma formation, and bile duct damage [25]. Immunized mice also showed increased levels of anti-mitochondrial antibody (AMA) production [25]. That group suggests that iNKT cells contribute to the perpetuation of PBC following an initial loss of tolerance to PDC-E2 and that iNKT cells play a critical role in PBC recurrence following liver transplantation [25].

## 8. Primary Sclerosing Cholangitis

Limited data have been obtained regarding the role of iNKT cells in PSC, despite the fact that cholangiocytes express CD1d and present lipid antigens to NKT cells [162]. In one model of ulcerative colitis with cholangitis in CD1 mice given 2.0% dextran sulfate sodium, researchers found decreased IL-4 levels and increased IFN-*γ*, with increased numbers of CD4+CD8+ cells in the liver but not in the colon. [163]. The same group of researchers analyzed numbers, surface markers, and cytokine production of mononuclear cells in the mouse model of cholangitis, which had also been given *α*-GalCer [164]. There was increased survival and weight gain noted in the *α*-GalCer treated mice, with decreases in IFN-*γ* release, CD4/CD8 ratio, and NK and NKT cells populations [164]. Those authors suggest that NKT cells treated with *α*-GalCer may promote a reduction in Th1 and an increase in Th2 cytokine activity [164]. Additionally, in NOD.c3c4 mice that spontaneously develop biliary inflammation in extrahepatic and intrahepatic bile ducts, iNKT cells were more abundant and displayed an activated phenotype [27]. Activation or blocking of NKT cells with *α*-galactosylceramide or anti-CD1d antibody injections, however, did not affect the biliary phenotype of NOD.c3c4 mice.

## 9. Vitamin D and iNKT Cells Function: A Role in Autoimmune Liver Disease?

As noted by several studies above, iNKT cells activation or suppression may induce an alteration in the cytokine milieu, in a direction that is either proinflammatory or anti-inflammatory, which appears to be disease-dependent. If the immunomodulatory properties of these cells are validated, they could become the target of novel therapeutic interventions. The question remains as to what therapeutic agents may be used in these conditions, if the notion that their immunomodulatory properties are therapeutic holds true. Recent studies have examined the role of vitamin D in immunomodulation [143], including the development and regulation of iNKT cells [91, 143–146]. Indeed, iNKT cells and CD4/CD8 intraepithelial lymphocytes are developmentally and functionally dependent on sufficient levels of vitamin D [147]. There has been ongoing research into the role of vitamin D and vitamin D deficiency in the development of autoimmune disease [44, 165, 166]. Multiple studies have noted the rising incidence of autoimmune disease with increasing distance from the equator, which has led to speculation that vitamin D deficiency may play a role in immunomodulation. Interestingly, multiple studies have noted vitamin D deficiency as well as vitamin D receptor (VDR) mutations in patients with autoimmune diseases (well reviewed in [166]), the most notable of which (mutations) are documented in MS [44, 166–169]. A relatively recent review also highlights the potential role the vitamin D deficiency likely plays in the development of AiLD [44]. ApaI polymorphism of the vitamin D receptor has been also recently shown to affect health-related quality of life in patients with primary sclerosing cholangitis [170]. Low vitamin D levels are also found to be common in patients with PBC and correlated with advanced disease, lack of response to UDCA therapy, and autoimmune disease comorbidity [45]. This alluded to the plausible scenario for a significant role for vitamin D as a prognostic marker of the severity of PBC and possibly the severity of other AiLDs. Recent studies have examined the role of vitamin D in immunomodulation [171], including the development and regulation of iNKT cells [94, 171–174]. Indeed, iNKT cells and CD4/CD8 intraepithelial lymphocytes are developmentally and functionally dependent on sufficient levels of vitamin D [175].

Animal models found that vitamin D is required in utero for normal iNKT cells development, with subsequent treatment (such as during clinically evident autoimmune disease) having little benefit [94, 174, 176]. The mechanisms underlying this have been well defined in studies examining the development of iNKT cells and the effect of vitamin D deficiency, as well as that of VDR knockout. The development of iNKT cells begins in the thymus, where they arise from conventional CD4+/CD8+ (double positive or DP) T cells [177]. These early TCR-positive iNKT cells are DP^dim^ and CD24+ and undergo rapid expansion at this stage [47]. Yu and Cantorna [174] found that, in subjects with adequate vitamin D levels, 91% of DP^dim^ iNKT cells go on to become mature CD24- iNKT cells, with most (61%) apoptotic cells being CD24+. In vitamin D deficiency, only 60% go on to become the CD24- mature type, with equal rates of apoptosis between CD44+ and CD24- cells [174]. Further maturation from CD44-NK1.1- to CD44+NK1.1- occurs in thymic precursors, which is then followed by CD44+NK1.1+ iNKT cells development [177, 178]. Interestingly, vitamin D knockout mice harbor iNKT cells blocked at the CD44+NK1.1- stage [94, 173, 174]. These iNKT cells were functionally defective with regard to the amount of cytokine secretion [173]. Cytokine deficiency and low iNKT cells numbers characterize VDR knockout mice, in contrast to vitamin D-deficient mice that only have decreased iNKT cells numbers but preserved IL-4 and IFN-*γ* producing function [174]. Recently, protective effects of 1,25-dihydroxyvitamin D3 in experimental autoimmune encephalomyelitis in mice have been attributed to the presence of NKT cells [179].

Although the effects of vitamin D on iNKT cells development have been elucidated, it is not clear as to whether vitamin D deficiency (or VDR knockout) results in a proinflammatory or anti-inflammatory state. In models of asthma and lung inflammation, abnormal iNKT cells number and function due to VDR knockout have been shown to ameliorate the disease [94, 180–183]. VDR knockout mice are unable to generate airway inflammation due to failed iNKT responses, with VDR knockout mice having decreased iNKT cells [94]. Also, Th2 cells in VDR knockout mice (with the C57BL/6 background) produced less IL-4, a reduction also found in iNKT cells (with BALB/c and C57BL/6 backgrounds) [92]. Additionally, iNKT cells were unable to produce IL-5 and IL-13 (BALB/c background), as well as IL-17 (C57BL/6 background) [94]. However, vitamin D deficiency and consequent abnormal iNKT cells numbers have been suggested to contribute to MS development [184–189], as well as hastening the clinical course of EAE [184]. Vitamin D ameliorates EAE, reduces the Th1 and Th17 cell response, and increases the Treg population [190–192]. A study by Torkildsen and colleagues [176] demonstrated that three patients with vitamin D-dependent rickets went on to develop MS, despite vitamin D supplementation, which adds to the debate on the effectiveness of early (in utero) vitamin D supplementation versus later treatment during clinical disease.

Recent research on vitamin D receptor- (VDR-) dependent signaling suggests that VDR functions to constrain the inflammatory response by targeting the miRNA-155-SOCS1 (suppressor of cytokine signaling 1) axis. The VDR-miRNA-155-SOCS1 pathway was investigated in the context of the autoimmune response associated with PBC. VDR/miRNA-155-modulated SOCS1 expression was decreased in PBC, leading to insufficient negative regulation of cytokine signaling [193]. 1,25-(OH)(2)-vitamin D(3) also prevented activation of hepatic stellate cells in vitro and ameliorated inflammatory liver damage but not fibrosis in the Abcb4(-/-) murine model of inflammation-induced cholestatic liver injury, fibrosis, and cancer [194].

## 10. Conclusion

The role of iNKT cells in autoimmune disease appears to be multifaceted, with these cells being involved not only in shaping the cytokine environment to be either Th1 or Th2 predominant but also in influencing B cell function and autoantibody production. Whether iNKT cells exert a proinflammatory or anti-inflammatory function varies between autoimmune diseases. The role of iNKT cells in AiLD remains to be elucidated. With the characterization of the functional phenotype of iNKT cells in AiLD and their relationship with disease activity, researchers may be able to establish immunomodulatory therapies to reduce the severity of disease or halt its progression. The immunomodulatory role of vitamin D is intriguing and appears to be highly relevant in this context, further underlying the need for more research.

## Figures and Tables

**Table 1:** Summary of studies in murine models supportive of a significant role of iNKT cells in the pathogenesis of AiLD.

**Model**	**iNKT cells number**	**iNKT cells cytokine**	**Liver injury**	**References**
Con-A-treated mice	↑	IFN-*γ*↑ IL-4 ↑ TNF-*α*↑	↑	[11–13]

Con-A-treated mice CCL2/MCP-1 neutralized	↑	IFN-*γ*↓ IL-4 ↑ TNF-*α*↓	↑	[14]

Con-A-treated CD1-deficient mice	(-* *-)	(-* *-)	↓	[15]

Con-A-treated SOCS1 cKO mice	**NS**	IFN-*γ ***ND** IL-4 **ND**	↑	[16]

Con-A-treated SOCS3 cKO mice	**NS**	IFN-*γ*↑ IL-4 ↑	↑	[17]

Con-A-treated PKC-*θ*(-/-) mice	↓	IFN-*γ*↓ TNF-*α*↓	↓	[18]

Con-A-treated Parp2(-/- )mice	↓	IFN-*γ ***ND**	↓	[19]

Con-A or CCl(4)- treated mice	↑	IL-33 ↑ (hepatocytes)	↑	[20]

Con-A-treated tpl2(−/−) mice	↓	IFN-*γ*↓ IL-4 ↓	↓	[21]

Con-A-treated PGAM5(−/−) mice	↓	IFN-*γ*↓ TNF-*α*↓	↓	[22]

Ripk3(-/-) mice	↓	IFN-*γ*↓ TNF-*α*↓	↓	[23]

dnTGF-*β*RII mice	↑	IFN-*γ*↑	↑	[24]

Xenobiotic-induced C57BL/6 mice and CD4 and CD8 KO mice	↑	IFN-*γ*↑ IL-4 ↑	↑	[25, 26]

NOD.c3c4 mice	↑	**NS**	↑	[27]

↑: increased; ↓: decreased.

(-* *-): absent; **NS**: not specified; **ND**: not different from WT.

## Abbreviations

AIH: Autoimmune hepatitis
*α*-GalCer: Alpha-galactosylceramide
AiLD: Autoimmune liver disease
Bregs: Regulatory B cells
DN: Double negative
DP: Double positive
EAE: Experimental autoimmune encephalitis
iNKT: Invariant natural killer T cells
NK: Natural killer cells
NKT: Natural killer T cells
MS: Multiple sclerosis
PBC: Primary biliary cholangitis
PSC: Primary sclerosing cholangitis
SLE: Systemic lupus erythematosus
TCR: T cell receptor
VDR: Vitamin D receptor.
